# Integrin Ligation Results in Nephrin Tyrosine Phosphorylation *In Vitro*

**DOI:** 10.1371/journal.pone.0148906

**Published:** 2016-02-05

**Authors:** Rakesh Verma, Madhusudan Venkatareddy, Anne Kalinowski, Sanjeevkumar R. Patel, Puneet Garg

**Affiliations:** Division of Nephroloigy, Department of Internal Medicine, University of Michigan School of Medicine, Ann Arbor, Michigan, United States of America; King's College London, UNITED KINGDOM

## Abstract

Nephrin is expressed at the basolateral aspect of podocytes and is an important signaling protein at the glomerular slit diaphragm. *In vitro* studies have demonstrated that Nephrin phosphorylation-dependent signaling is able to assemble a protein complex that is able to polymerize actin. However, proximal signaling events that result in nephrin tyrosine phosphorylation are not well understood. Nephrin deletion in mice and human nephrin mutations result in developmental failure of the podocyte intercellular junction resutling in proteinuria. This has been presumed to be due to a failure to respond to an external polarized cue in the absence of nephrin or a failure to transduce an outside-in signal in patients with nephrin mutations. The nephrin extracellular domain binds to itself or neph1 across the foot process intercellular junction. Nephrin is tyrosine phosphorylation-silent in healthy glomeruli when presumably the nephrin extracellular domain is in an engaged state. These observations raise the possibility of an alternate proximal signaling mechanism that might be responsible for nephrin tyrosine phosphorylation. Here we present data showing that integrin engagement at the basal aspect of cultured podocytes results in nephrin tyrosine phosphorylation. This is abrogated by incubating podocytes with an antibody that prevents integrin β1 ligation and activation in response to binding to extracellular matrix. Furthermore, nephrin tyrosine phosphorylation was observed in podocytes expressing a membrane-targeted nephrin construct that lacks the extracellular domain. We propose, integrin-activation based signaling might be responsible for nephrin phosphorylation rather than engagment of the nephrin extracellular domain by a ligand.

## Introduction

Podocytes are highly specialized terminally differentiated epithelial cells that are an important component of the selective filtration barrier of the renal glomerulus. The podocyte intercellular junction or slit diaphragm is a modified adherens junction. Several unique junctional proteins like nephrin and neph1 have been identified at the slit diaphragm and are responsible for formation as well maintenance of the filtration barrier [1,2]. Nephrin when tyrosine phosphorylated assembles a protein complex that is able to regulate actin cytoskeletal dynamic [3–6]. In experimental conditions, investigators have employed artificial means to phosphorylate nephrin due to lack of a physiological nephrin ligand. A popular strategy has been adapted from immunological studies, where “clustering” of membrane receptors using antibodies results in tyrosine phosphorylation of the cytoplasmic domain of the protein [7]. Though it has been a successful strategy to identify signaling events that occur as a consequence of nephrin phosphorylation [4–6,8], it is unlikely that this occurs *in vivo*. It is not surprising that observations made by artificial clustering of nephrin have provided support to the hypothesis of an activating nephrin ligand. Interestingly, both *in vitro* and *in vivo* studies have shown that nephrin is not phosphorylated at its basal steady state [6,9,10]. In mature healthy glomeruli, nephrin is predominantly unphosphorylated when it is presumably in contact with its extracellular ligand [6,9,11]. Tyrosine phosphorylation of nephrin was reported to be decreased when it *trans*-interacted with Neph3 in cell culture [11]. An increase in nephrin phosphorylation on Y1191 and Y1208 has been reported in protamine sulfate model of podocyte injury and during glomerular development in mice [6,9]. The Src family kinase Fyn has been shown to be responsible for nephrin phosphorylation on multiple tyrosine residues including the residues that are important for nephrin-nck interaction and actin polymerization [10,12]. Emerging data suggests that there is a close signaling relationship between the focal adhesion complex and the nephrin receptor complex [9]. Though there are several mechanisms that result in an increase in Src kinase activity, integrin activation is a well-described mechanism that results in activation of both Src and FAK family kinases [13–18]. Based on integrin’s ability to activate Src family kinase activity following ligation [13,14], we hypothesized that integrin ligation-dependent Src kinase activation results in nephrin tyrosine phosphorylation.

**Fig 1:**
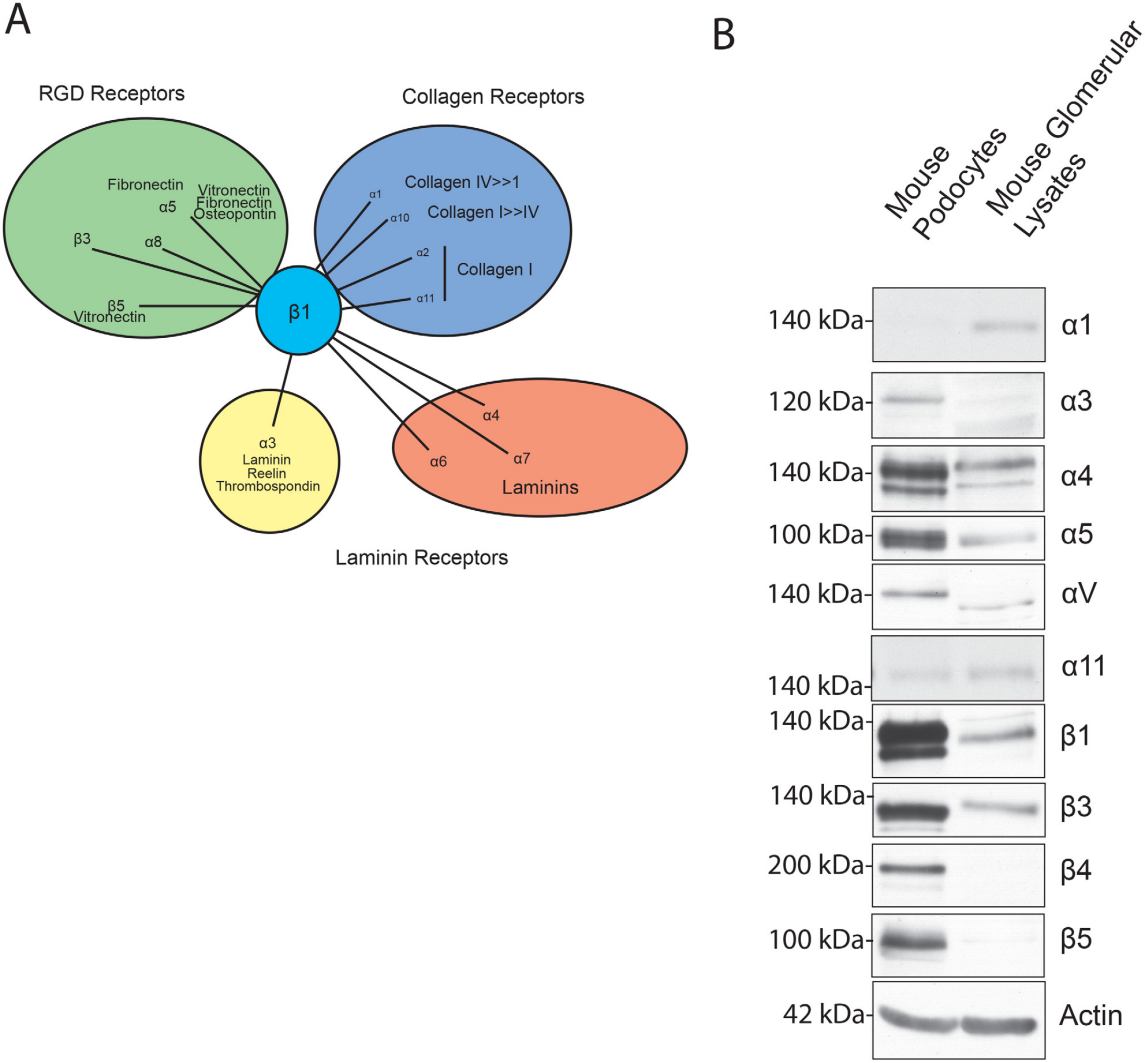
(A) Schematic showing integrin chains and receptor specificity of integrin heterodimers. β1 integrin is common to many of the heterodimers that bind to laminin, fibronectin and collagen. (B) Integrin chain expression profile of cultured mouse podocytes cells and mouse whole glomerular lysate.

Integrins are transmembrane heteromeric receptors that mediate interactions between cells as well as between cells and the extracellular matrix (ECM). Integrins are classified as laminin, collagen and arginine-glycine- aspartic acid (RGD)- binding receptors based on their heterodimeric composition that provides specifiity to binding to different ECM components (Fig 1A) [19]. Studies examining cell matrix binding proteins that are expressed in the kidneys found α3β1 integrin to be highly expressed in the glomeurlus [20–24]. Genetic deletion of either α3 or β1 integrin in mouse podocytes results in podocyte developmental abnormalities [22,25–27]. Identifying the signaling mechanisms that result in nephrin tyrosine phosphorylation are crucial to better understand the cytoskeletal changes that occur both during development and injury and fills an important gap in our present knowledge. Here we present data that shows in an *in vitro* model, integrin ligation and activation results in nephrin tyrosine phosphorylation when cultured podocytes are plated on a surface coated with laminin or fibronectin. The specificity of this proposed integrin-nephrin signaling is demonstrated by abrogation of nephrin phosphorylation when ligation of β1 and β3 integrin was inhibited. The proposed signaling mechanisms provide an alternate model of nephrin phosphorylation that is consistent with the observations made both *in vivo* and *in vitro*.

## Experimental Procedures

### Plasmids

Plasmid encoding full-length mouse nephrin has been described previously [3,4,28]. Plasmid encoding membrane targeted nephrin cytosplasmic domain (myr-Nphs1^ΔECD/TM^) was generated by sub-cloning mouse nephrin cytoplasmic domain into pHom-mem1 vector (Cat# 635064) obtained from Clontech. DsRED-CrkI plasmid was a kind gift from Dr. Mochizuki (National Cardiovascular Center Research Institute, Osaka, Japan) [29]. All plasmid sequences were verified by DNA sequencing and restriction digestion.

### Antibodies

Purified rabbit polyclonal antibodies against nephrin (31) and p-nephrin (recognizes phosphorylation on mouse nephrin Y1191, 1208 residues) (4) were previously described. Antibodies against FAK and phospho-FAK Y397, *p-Src* Y416 and Fyn were obtained from Cell Signaling Technology (Danvers, MA). Activated β1 integrin antibody (HUTS4) was obtained from EMD Millipore (Bedford, MA). Antibodies against various integrin subunits (α4, α5, αV, β1, β3, β4 and β5) were obtained as a sampler pack (#4749) from Cell Signaling Technology (Danvers, MA). The Integrin α3 antibody (P1B5) monoclonal antibody [30] developed by E.A. Wayner and W.G. Carter from Fred Hutchinson Cancer Research Center (Seattle, WA) as well as β1 integrin blocking monoclonal antibody (AIIB2) [31] developed by Caroline Damsky (UCSF, San Francisco, CA) were obtained from the Developmental Studies Hybridoma Bank, created by the NICHD of the NIH and maintained at The University of Iowa, Department of Biology, Iowa City, IA. Integrin α1 (ab34445) and α11 (ab114113) antibodies were obtained from Abcam (Eugene, OR). Integrin β3 blocking antibody (B3A) was obtained from Millipore. Monoclonal antibody against actin (AC-15) was obtained from Sigma (St. Louis, MO).

### Immunoblotting

Proteins were extracted from plasma membranes in RIPA buffer (PBS containing 0.1% SDS, 1% Nonidet P-40, 0.5% sodium deoxycholate and 100mM potassium iodide). Lysates were resolved using SDS-PAGE and transferred to PVDF membrane (GE Healthcare) using semi-dry transfer (Bio-Rad). Membranes were blocked using 5% milk or 5% BSA (phospho proteins). Immunoblotting for activated β1 integrin was done under non-reducing conditions. Immunoblotting was performed with the indicated primary antibody followed by HRP-conjugated secondary antibody of the appropriate species.

### Generation of immortalized podocyte cell line

To isolate glomeruli, mice were perfused through the heart with magnetic 4.5μm diameter Dynabeads (Life Technologies) at 8 x 10^7^ dilution in PBS. The kidneys were removed and minced into 1 mm cubes and digested with collagenase (1 mg/ml collagenase A in 100 U/ml deoxyribonuclease I in HBSS) at 37°F for 30 minutes with gentle agitation. The collagenase-digested tissue was gently pressed through a 100μm sieve using a flattened pestle. The filtered cells were passed through a new strainer and collected. The cell suspension was centrifuged at 200 X g for 5 minutes. The supernatant was discarded and the cell pellet was resuspended in HBSS. The dynabead containing glomeruli were isolated using a magnet and washed at least three times with HBSS. The tissue was kept over ice throughout the procedure except for the initial incubation with collagenase. The protocol has been described in detail by Takemoto et al [32]. This method provides a highly pure glomerular preparation with purity close to 98%. Isolated glomeruli were plated onto 10 cm culture dishes. Cell outgrowths from the glomeruli were seen in 3 days and were primarily podocytes [33,34]. The cells were trypsinized and plated on new culture plates. When nearing confluence, cells were treated with adenovirus carrying SV40 T-antigen (titer 1.3 X 1011; Viral Core, University of Michigan, Ann Arbor, MI). Packaged viral particles were dissolved in complete media at MOI = 200 and minimal volume covering the cells. 4 h later, media was added to a volume optimal for the dish size. Media was replaced 14 h later with fresh complete media. Even though endogenous nephrin expression in early passage cells was enough to be seen using Western blot, we generated a stable line cell line expressing full-length mouse nephrin using puromycin selection (Mspod^nphs1^).

### Cell Culture and transfection

Transient transfections were carried out in immortalized mouse podocyte cells cultured in RPMI with glutamax (Invitrogen) and supplemented with 10% FBS (Invitrogen Corp.) and 200 U/ml penicillin and streptomycin (Roche Applied Science) along with ITS (Insulin, Transferrin and Selenium) (Invitrogen). Transfections were performed using Lipofectamine 2000 (Invitrogen Corp.), Fugene HD (Roche) and electroporation using Amaxa nucleofactor II (Amaxa biosystem) as per manufacturer’s directions. Collagen 1 (4.73 mg/ml, #354236) or fibronectin (1 mg/ml diluted in PBS, #356008) were obtained from BD Biosciences, Bedford, MA. Laminin (0.5 mg/ml in TBS, #L6274) was obtained from Sigma-Aldrich; St. Louis, MO. 2–3 ml of laminin, collagen 1 and fibronectin was added to 6-well culture plates to completely coat the well- surface and incubated for 2 h at 37°C. Plates were then washed thoroughly with PBS prior to plating of cells in complete media.

### Integrin blocking experiments

β1 integrin blocking antibody (AIIB2), β3 integrin blocking antibody (B3A) or mouse monoclonal IgG (control) was mixed with 1 x 10^5^ cells in 300 μl of media at 10 μg/ml concentration for 60 minutes at ambient temperature prior to plating the cells on culture dishes. The cells were washed gently prior to addition of lysis buffer as they detached easily in the presence of β1 and β3 integrin blocking antibody.

### ImageJ quantitation Statistical Analysis

Data are presented as Mean ± SEM throughout the text unless otherwise specified. The number of experiments performed for each experiment has been mentioned in the figure legends. All experiments were performed at least 3 times. ImageJ software was used to quantify the density of protein bands on Western Blots. Statistical comparisons were performed using two-tailed *t* test and ANOVA where applicable. A value of P≤ 0.05 was considered to represent statistically significant difference.

### Animal Studies

All animal studies were approved by the university committee on the use and care of animals institutional review board at the University of Michigan School of Medicine. Animals were anaesthetized using a combination of ketamine and xylazine prior to perfusion. After the kidneys were removed the animals were euthanized by bilateral thoracotomy.

## Results

### Nephrin phosphorylation occurs following integrin ligation

**Fig 2:**
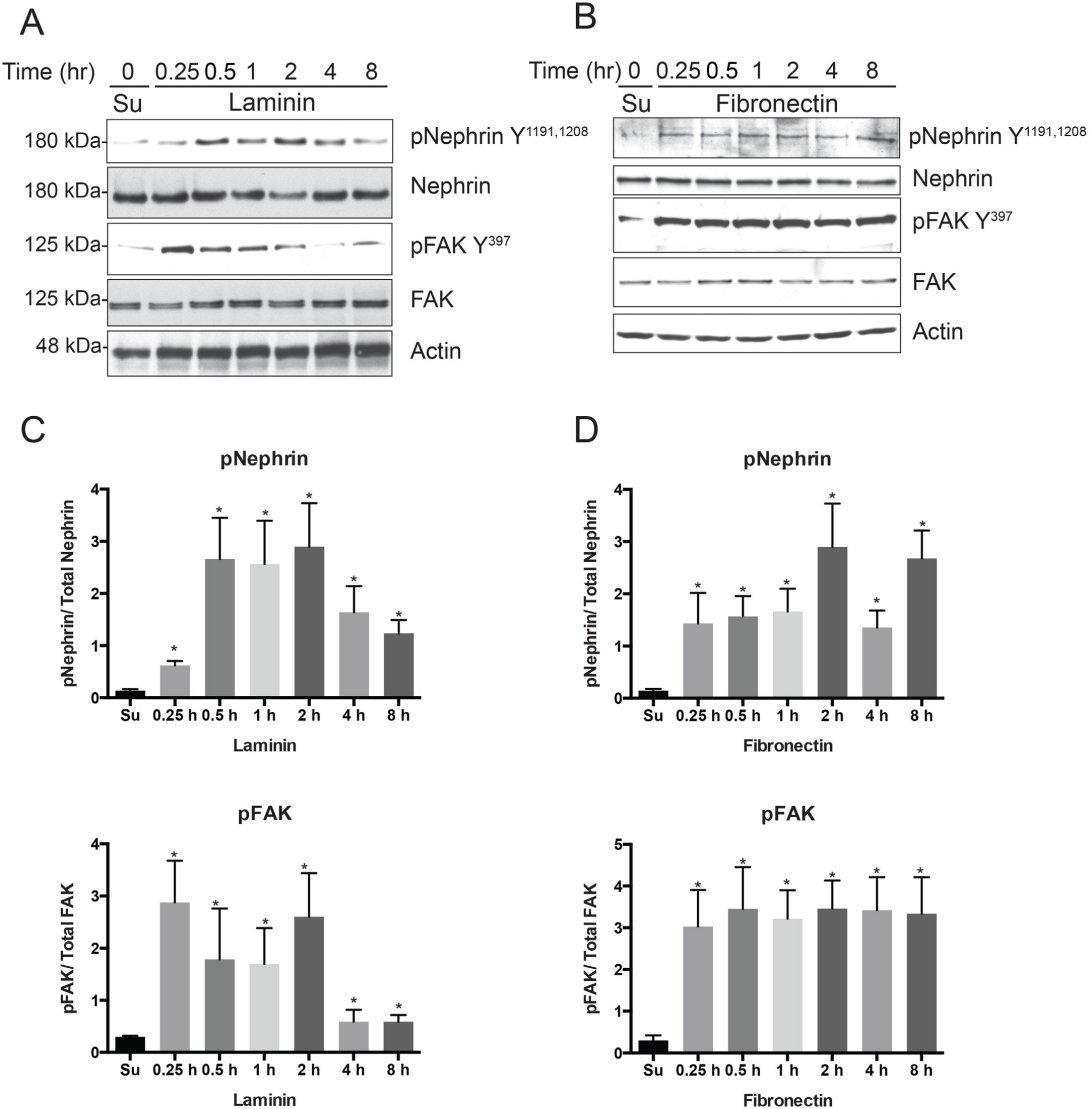
Nephrin is tyrosine phosphorylated when mouse podocytes are plated on laminin and fibronectin coated surface. Mouse podocytes were plated on culture dishes coated with laminin (A) and fibronectin (B) for the indicated time points. At time 0 cells were maintained in suspension (Su) on a rocker prior to plating. Cells were lysed at various time points and lysates were resolved using SDS-PAGE. Membranes were probed with the indicated antibodies. FAK Y397 phosphorylation was used as a surrogate for integrin activation. (C and D) Bands densitometry using ImageJ software for 4 separate experiments showing increase in nephrin tyrosine phosphorylation as well as FAK Y397 phosphorylaton at different time points. Data are mean values ± SEM. *P<0.001.

We first examined the expression of various integrin α and β chains in the primary mouse podocytes cell line generated by us as wells as in isolated glomeruli (Fig 1A). There is expression of alpha subunits α3, α4, α5 and αV and for beta subunits β1, β3, β4 and β5 in the mouse podocyte cell line that we used for the experiments (Fig 1B). Mouse glomerular lysate also had expression of these subunits but did not express β4 and β5 integrin subunits. Since glomerular lysate has a contribution from multiple cell types it may not reflect expression levels of β4 and β5 subunits specifically in podocytes because of dilutional effect. To test our hypothesis that nephrin will be tyrosine phosphorylated following integrin ligation we plated immortalized podocytes cells stably expressing nephrin (Mspod^nphs1^) on a surface coated with fibronectin and Laminin. To assess for integrin ligation/activation we used phospho-FAK Y397 antibody. FAK phosphorylation has been observed and widely used as a surrogate for integrin activation [35–38]. We observed an increase in FAK Y397 and Nephrin (Y1191, 1208) phosphorylation, when cells were plated on laminin or fibronectin coated wells (Fig 2A and 2B). We will refer to tyrosine phosphorylation of nephrin Y1191 and Y1208 cytoplasmic domain residues that are important for nephrin-nck interaction as nephrin tyrosine phosphorylation in the remaining text. Quantification of the bands using densitometry for phospho-nephrin shows a statistically significant rise in nephrin phosphorylation at the 15-minute time point following plating of cell on laminin-coated surface (Fig 2A and 2C). The increase in nephrin phosphorylation peaked at 30 minutes and showed a decline after the 2-hour time point. There is persistence of nephrin phosphorylation even 8 hours following plating of cells. Similarly, plating of cells on fibronectin also showed an increase in nephrin tyrosine phosphorylation that appears to persist longer and at a higher intensity when compared to laminin (Fig 2B and 2D). As anticipated there was increase in FAK^Y397^ phosphorylation in both conditions suggesting integrin activation. FAK phosphorylation was also quantified using image J densitometry and follows a pattern similar to phospho-nephrin under each condition. The fibronectin-coated surface resulted in persistence of both nephrin and FAK phosphorylation at higher intensity even at the 8-hour time point. We observed decline of both phosphorylated nephrin and FAK after the 2-hour time point when podocytes were plated on laminin coated surface (Fig 2C).

### Nephrin tyrosine phosphorylation is abrogated in the presence of β1 integrin monoclonal antibody

**Fig 3:**
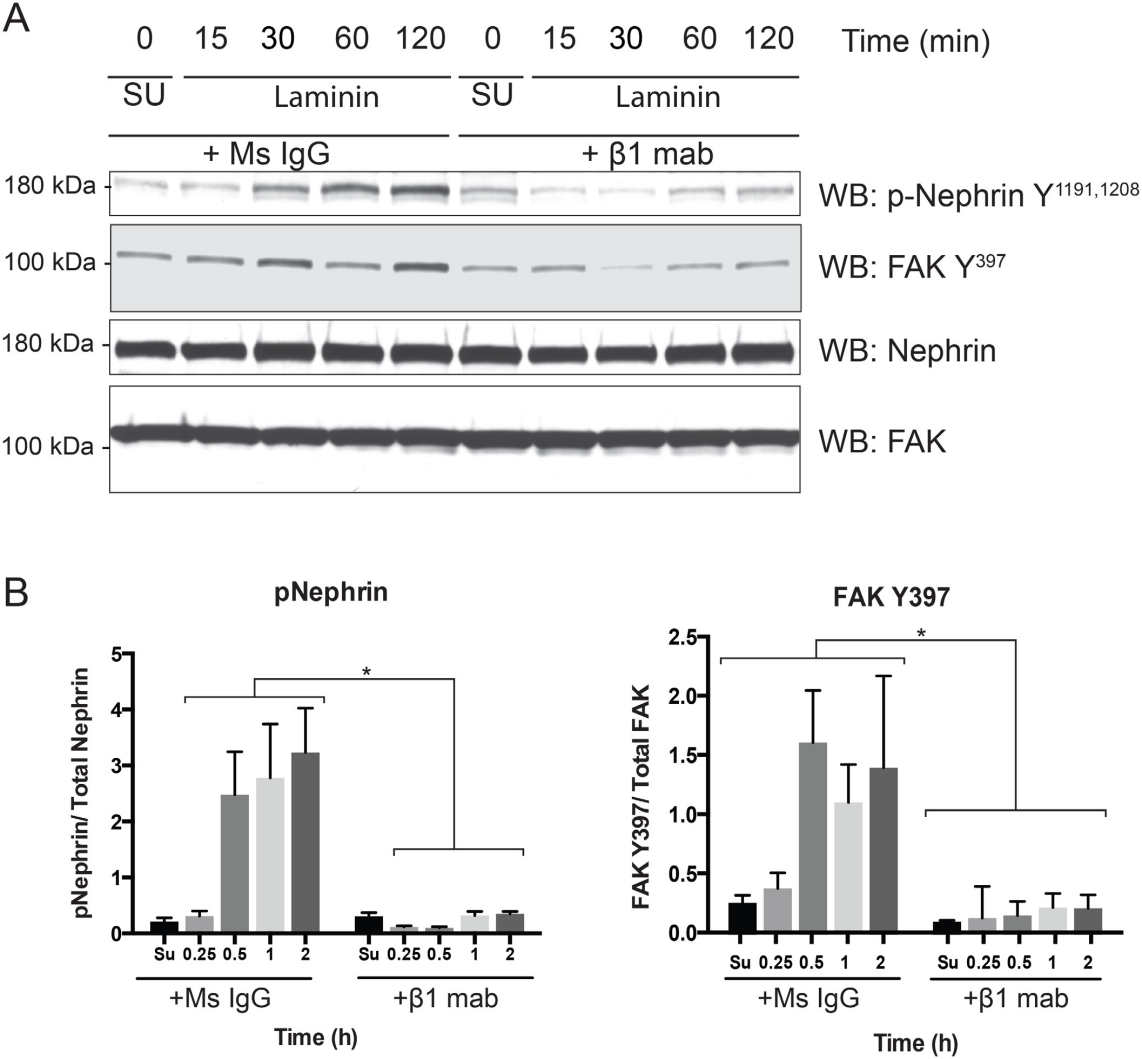
Nephrin tyrosine phosphorylation on laminin-coated surface is abrogated by pre-incubation of podocytes with β1 blocking antibody. (A). Mouse podocytes were incubated with β1 blocking mab and mouse IgG (control) prior to plating on laminin coated surface. Time 0 denotes cell in suspension (Su). Cells were lysed at various time points and lysates were resolved using SDS-PAGE. Membranes were probed with the indicated antibodies. FAK Y397 phosphorylation was used as a surrogate for integrin activation. (B) Bands densitometry using ImageJ software for 4 separate experiments showing increase in nephrin tyrosine phosphorylation as well as FAK Y397 phosphorylaton at different time points. Data are mean values ± SEM. *P<0.001, using two-tailed *t* test.

Since β1 is a common integrin subunit and has been shown to have a predominant role in podocyte development and homeostasis, we used a blocking antibody against integrin β1 to prevent interaction with laminin [31]. Studies have demonstrated a decrease in FAK activation (decreased FAK Y397 phosphorylation) in the presence of the β1 blocking antibody [39]. Podocytes (Mspod^nphs1^) were incubated with the β1 antibody or control (mouse IgG) while in suspension on a rocker for 60 minutes at ambient temperature prior to plating on laminin coated culture plates. We observed abrogation of nephrin phosphroylation when podocytes were incubated with β1 antibody prior to plating (Fig 3A). As anticipated there was decrease in FAK Y397 phosphorylation in the presence of the β1 blocking antibody. We did not observe any difference in cell attachment or spreading in the presence of β1 blocking antibody though the cells detached easily during the washing step. Quantification of band density using densitometry from 4 separate experiments is shown in Fig 3B. There is a statistically significant decrease (p value< 0.001) in both nephrin and FAK phosphorylation at all time points. These results suggest that integrin β1 ligation is necessary for nephrin tyrosine phosphorylation.

**Fig 4:**
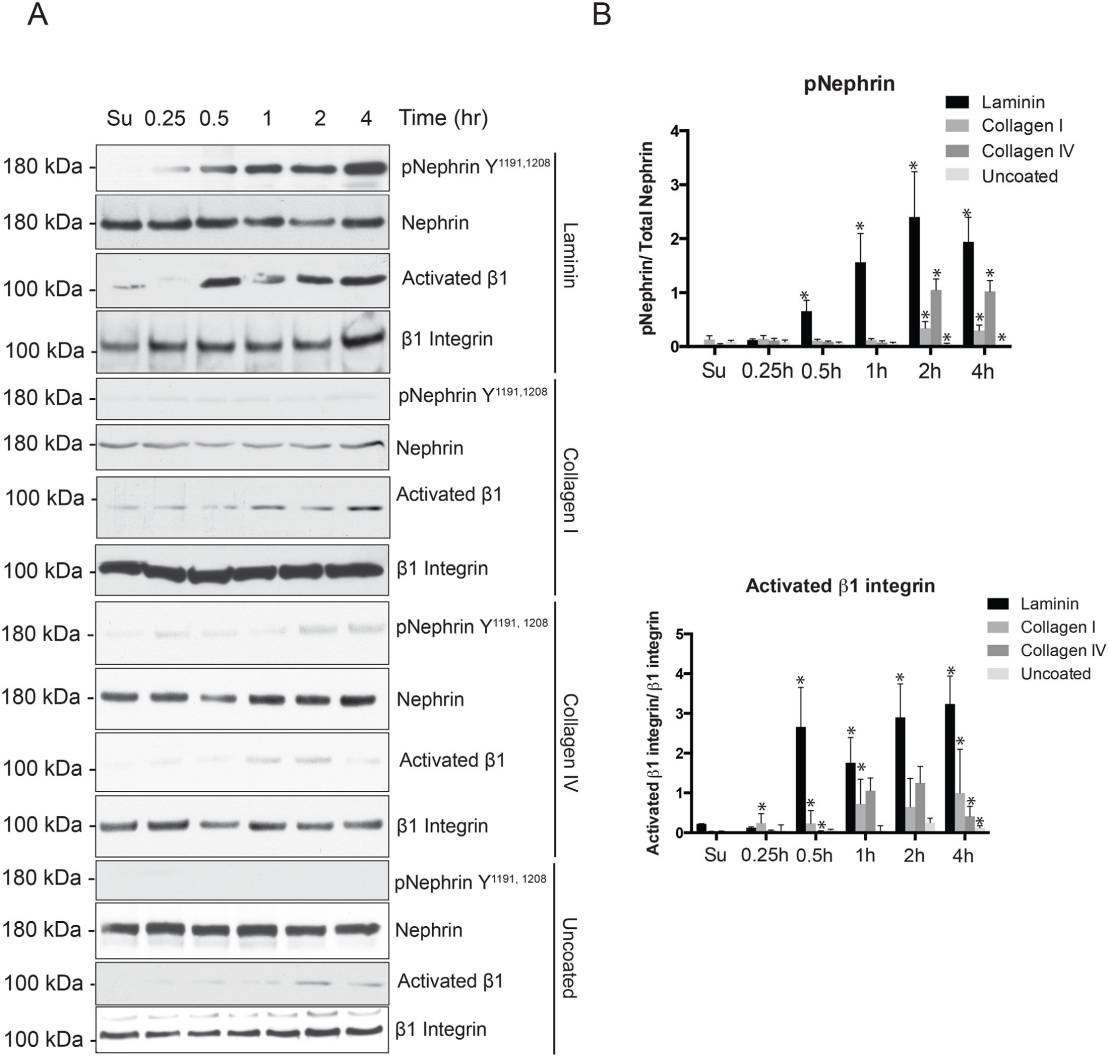
Nephrin tyrosine phosphorylation does not increase when mouse podocytes were plated on collagen-coated or uncoated surface. (A) Lysates of mouse podocytes plated on fibronectin-coated, collagen I and IV-coated and uncoated surface were blotted for phospho-nephrin and p-FAK. The increase in nephrin phosphorylation observed on plating on fibronectin coated surface was not seen when cells were plated on collagen-coated or uncoated surface. Antibody that detects activated β1 integrin (HUTS5) was used to to assess integrin activation under non-reducing conditions. (B) Bands densitometry using ImageJ software for 4 separate experiments showing increase in nephrin tyrosine phosphorylation as well as activated β1 integrin at different time points. Data are mean values ± SEM. *P<0.001.

*Nephrin tyrosine phosphorylation is not observed when cells are plated on collagen-coated or uncoated surface*. In order to examine whether nephrin phosphorylation was specific for the ECM component we used collagen I-coated, collagen IV-coated and uncoated surface. Compared to laminin, nephrin phosphorylation was low on collagen IV-coated surface but was hardly observed when cells were plated on collagen I-coated or uncoated surfaces (Fig 4A). To assess for integrin β1 ligation and activation we used activated β1 antibody (HUTS4) that recognizes specific epitopes (355–425 amino acids) on the common β1 subunit that undergoes conformational change following activation [40]. Increase in activated β1 integrin was seen following plating of cells on laminin. The increase in activated β1 integrin was singnificantly lower and was observed at a later time point when cells were plated on collagen-coated and uncoated surfaces. The integrin heterodimers containing α2 and α11 chains interact with collagen [19], and are not highly expressed in our cultured podocytes (Fig 1B). Quantification of bands using densitometry (ImageJ software) is shown in Fig 4B. These results suggest that nephrin phosphorylation requires specificity of signaling that originates from the cell-culture surface interaction and is not promiscuous. Cell-specific heterogeneous signaling responses following activation or ligation of various integrin heterodimers have been reported [41,42].

### Nephrin lacking the extracellular domain is tyrosine phosphorylated following plating on laminin- coated surface

**Fig 5:**
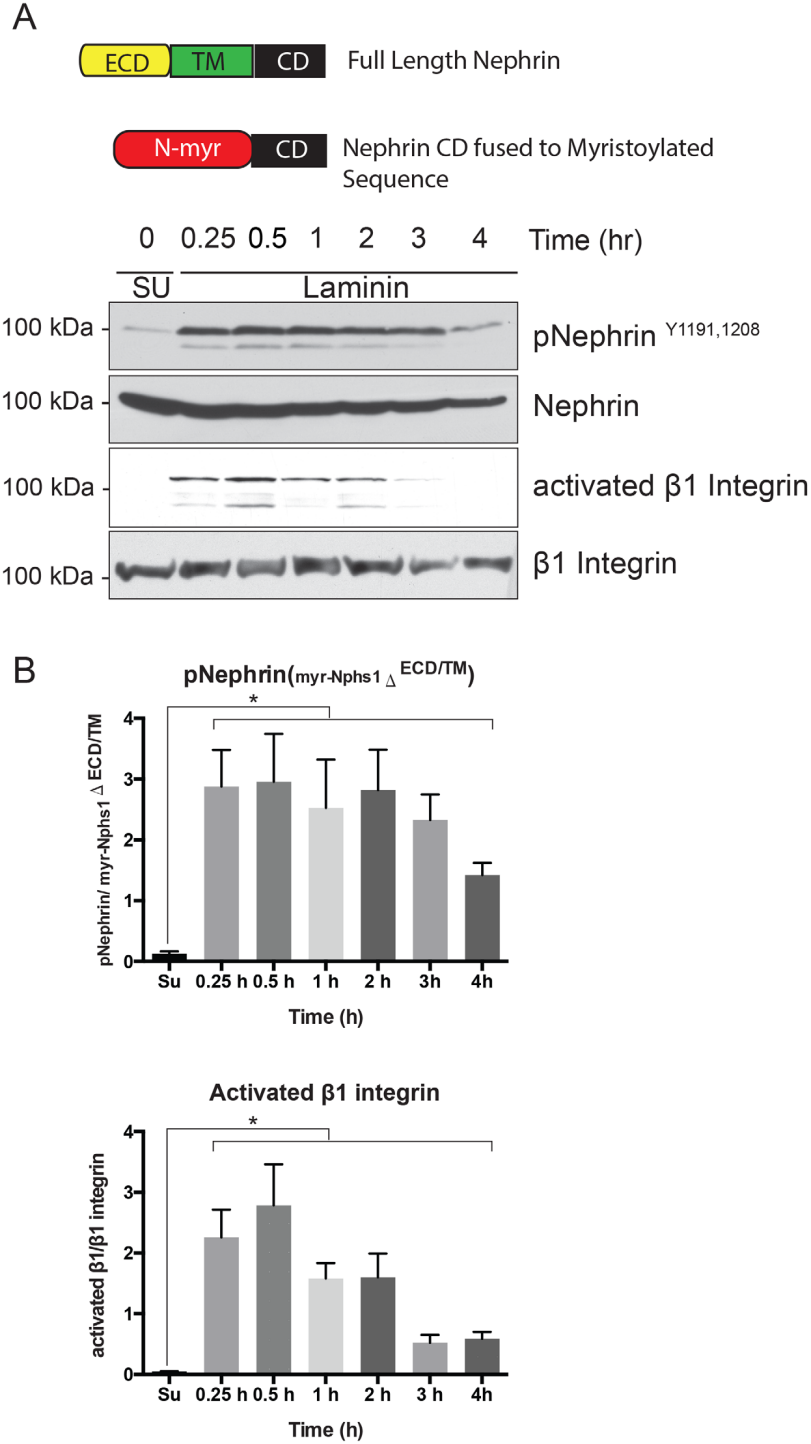
Nephrin extracellular domain is not required for nephrin tyrosine phosphorylation. (A). HEK 293 cells expressing a nephrin construct where the extracellular domain is substituted by a myristoylated sequence is tyrosine phosphorylated following plating of cells on laminin coated surface. (B) Band densitometry using ImageJ software for 4 separate experiments showing increase in nephrin tyrosine phosphorylation as well as activated β1 integrin at different time points. Data are mean values ± SEM. *P<0.001, using two-tailed *t* test.

Nephrin extracellular domain interacts with itself and with Neph1 across the foot process intercellular junction [8]. Activation of Nephrin extracellular domain cannot be excluded following plating of cells expressing full-length nephrin. We generated a construct where nephrin cytoplasmic domain is conjugated to a myristoylated sequence at its N-terminal (myr-Nphs1^ΔECD/TM^). The myristoylated sequence targets the expressed nephrin to the plasma membrane. HEK293 cells expressing myr-Nphs1^ΔECD/TM^ showed similar increase in Nephrin phosphorylation following cell plating on a laminin-coated surface (Fig 5A). We did not use podocytes for these experiments in order to avoid interference from endogenous nephrin or neph1. Quantification of bands using densitometry from 4 independent experiments is shown in fig 5B. The change in phosphorylation and activation of β1 integrin was statistically significant (p value <0.001) at all time points compared to control (suspension). These observations suggest a signaling mechanism that is independent of a ligand binding to the nephrin extracellular domain.

### Src kinase Fyn is necessary for integrin-dependent nephrin tyrosine phosphorylation

**Fig 6:**
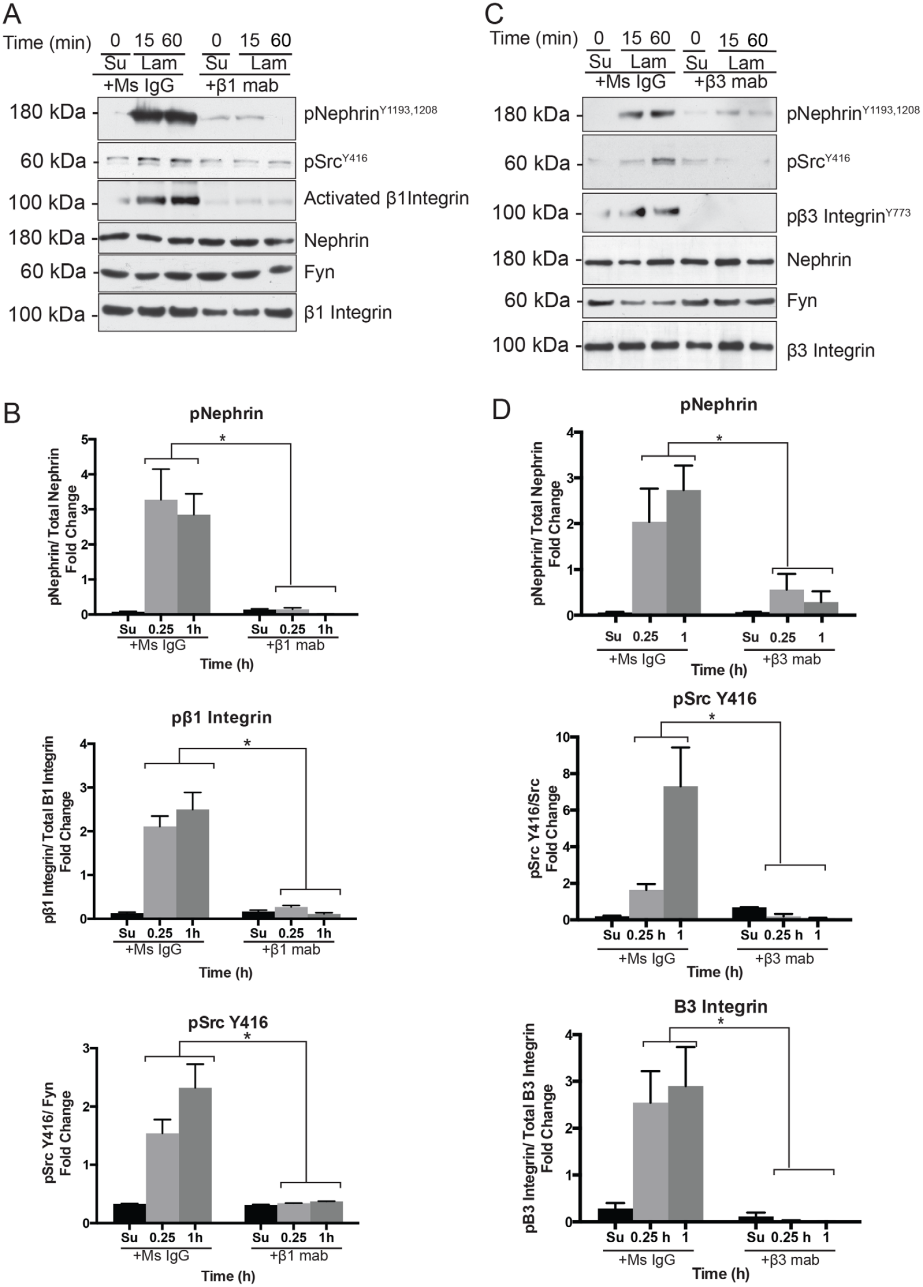
Src activation is abrogated in the presence of β1 blocking mab. (A) Cultured mouse podocyte lysates show decrease in Src Y416 phosphorylation in the presence of β1 blocking mab suggesting decreased activation of Src kinases when integrin β1 activation is prevented. Mouse IgG was used as control. (B) Bands densitometry using ImageJ software for 4 separate experiments showing increase in nephrin and Src Y416 tyrosine phosphorylation, as well as activated β1 integrin at different time points. Data are mean values ± SEM. *P<0.001, using two-tailed *t* test.

**Fig 7:**
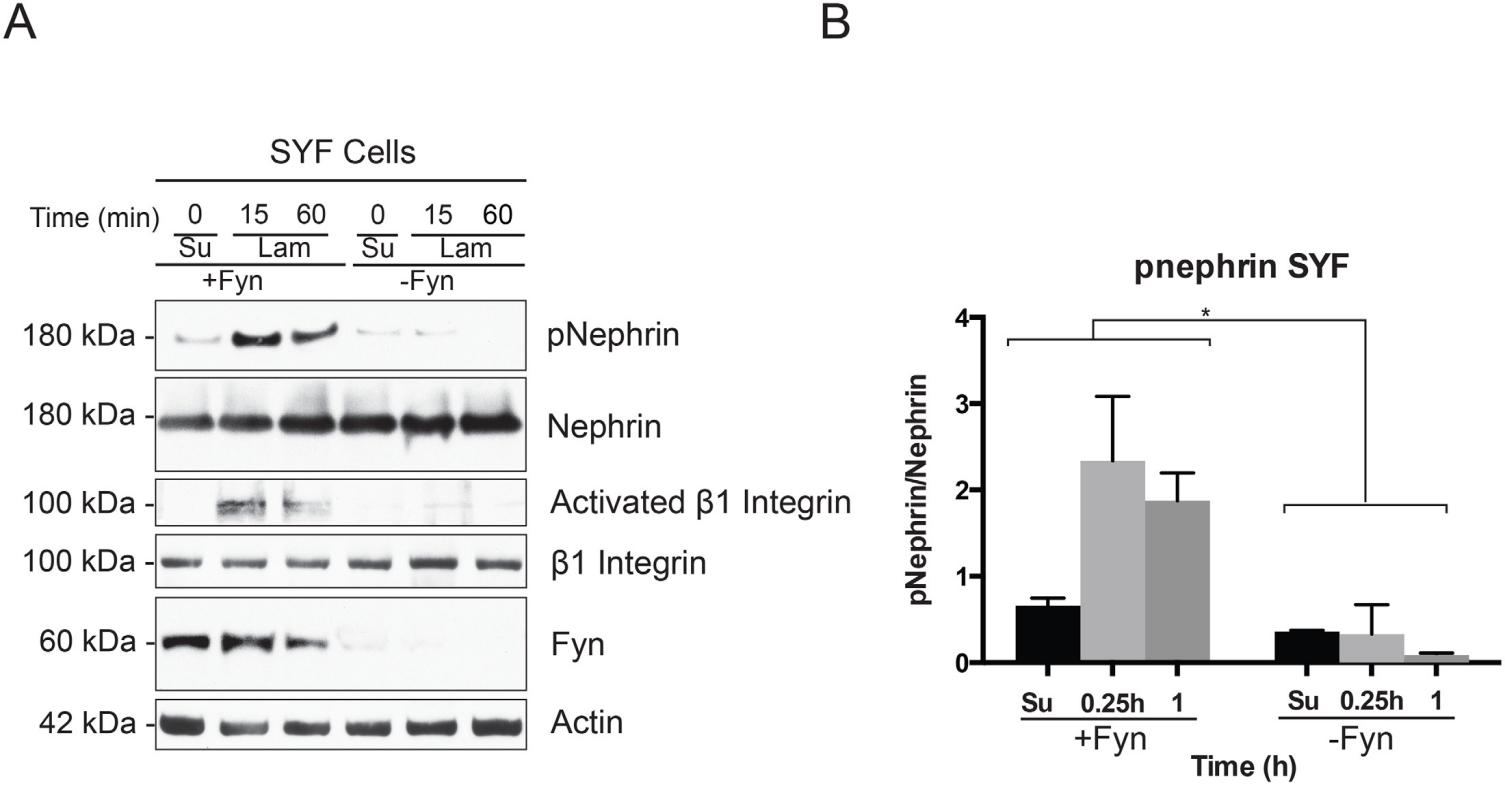
Src Kinase Fyn is necessary for nephrin phosphorylation. (A) SYF cells that lack Src, Fyn and Yes were transfected with full-length nephrin. Cells were plated on laminin-coated surface for indicated time points. Nephrin phosphorylation as well as β1 integrin activation was observed only after introduction of Fyn in SYF cells. (B) Band densitometry using ImageJ software for 4 separate experiments showing increase in nephrin tyrosine phosphorylation in the presence and absence of Fyn at different time points. Data are mean values ± SEM. *P<0.001, using two-tailed *t* test.

Integrin activation specifically β1 integrin ligation is associated with activation of focal adhesion associated kinases like Src and FAK [15,16,43–45]. We examined whether Src kinase activation occurred following plating of cells on laminin. Podocytes (Mspod^nphs1^) in suspension were incubated with β1 monoclonal antibody or mouse IgG on a rocker for 60 minutes at room temperature prior to plating. Cells were then plated on laminin coated surface for the indicated time points. There was an increase in Src Y416 phosphorylation following plating of cells on laminin, which was abrogated when cells were pre-incubated with the integrin β1 antibody (Fig 6A). Src Y416 phosphorylation suggests activation of Src kinase [46,47]. Activated β1 integrin as well as nephrin phosphorylation was also reduced in the presence of the integrin β1 blocking antibody. Quantification of band density using Image J software from 4 separate experiments are shown in Fig 6B. Similar results were obtained when β3 integrin blocking antibody was used (Fig 6C and 6D). In order to test the necessity of Src kinase in nephrin phosphorylation following plating on laminin-coated surface, we transfected full-length nephrin in SYF cells that lack Src, Yes and Fyn. In the absence of Src, Yes and Fyn, nephrin phosphorylation was not observed following plating of cells on laminin (Fig 7A). The expected increase in nephrin phosphorylation was observed once Fyn was re-introduced in SYF cells. Integrin β1 phosphorylation or activation was also abrogated in the absence of Fyn. Quantification of nephrin phosphorylation in the presence and absence of Fyn was perfromed using densitometry (Fig 7B). These observations suggest that integrin- mediated Src activation at the basolateral surface might be responsible for nephrin tyrosine phosphorylation.

### Activated β1 integrin and phosphorylated nephrin co-localize during glomerular development and in vitro

**Fig 8:**
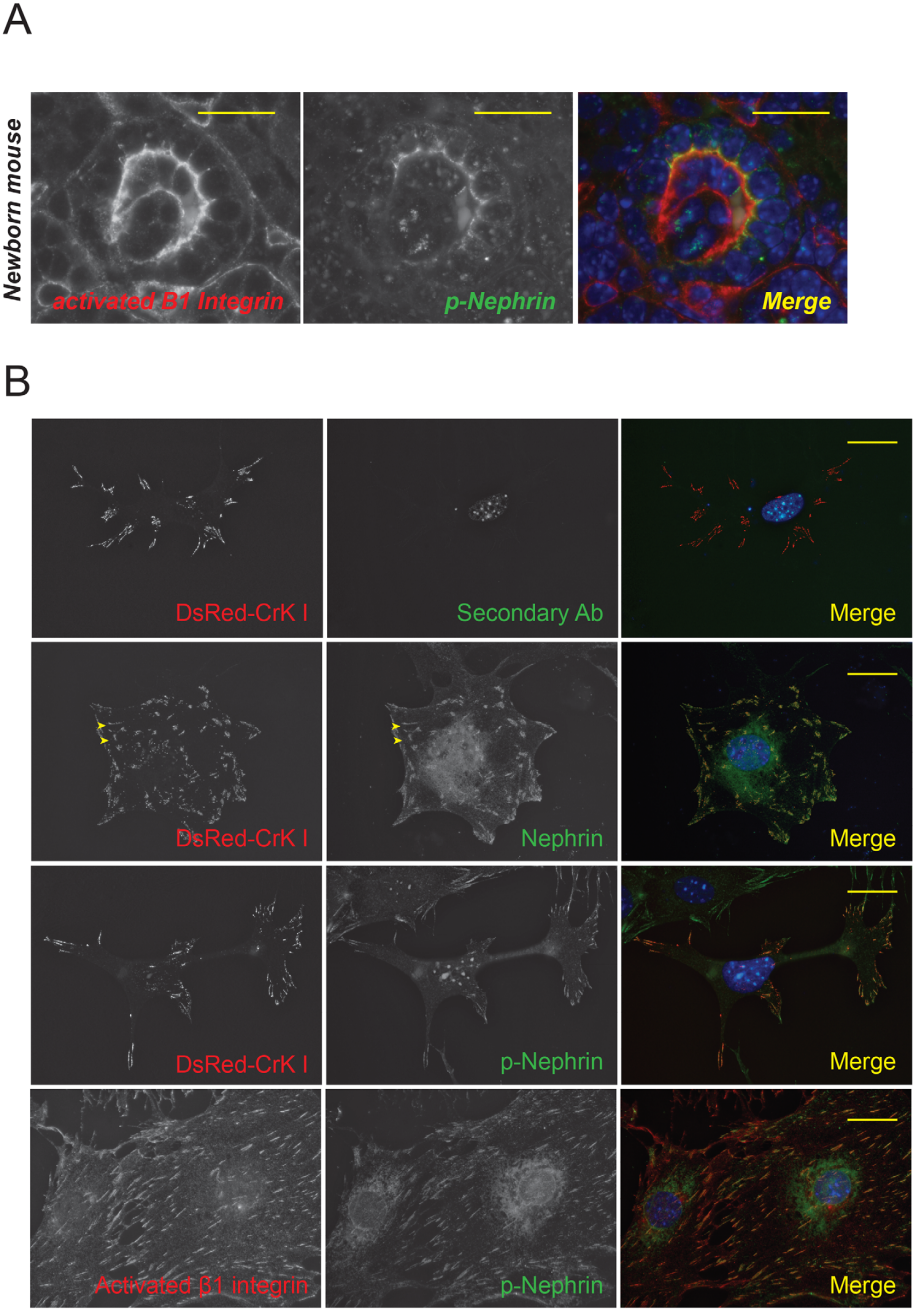
(A) **Activated β1 integrin and phospho-nephrin colocalize in developing glomeruli**. Frozen sections from newborn mouse kidneys were immunstained with activated β1 integrin and phospho-nephrin antibodes showing colocaliztion during glomerular devlopment. (B) Endogenous nephrin is seen in focal adhesions in cultured podocytes. Mouse podocytes expressing DsRED tagged CrkI (found in focal adhesions) were plated on laminin-coated glass cover slips. Immunostaining for nephrin shows presence of nephrin at the leading edge as well as in focal adhesions (Panel B). There is co-localization of CrkI and activated β1 integrin with phosphorylated nephrin (Panel C and D). Panel A shows the specificty of staining as the secondary antibody by itself does not give a signal. Scale bars, 20μm.

In order to examine the physical proximity of both integrin and nephrin *in vivo*, we examined newborn mouse kidneys. We hypothesized that during development both integrin activation and nephrin phosphorylation would be observed in developing glomeruli. Newborn mouse kidney sections were immunostained with p-nephrin (Y1191 and Y1208) as well as activated β1 integrin antibodies (Fig 8A). We observed co-localization of both p-nephrin and activated β1 integrin in developing glomeruli. Similarly, endogenous nephrin colocalizes with DsRed-labeled Crk1 in cultured mouse podocytes (Fig 8B, see arrows). Crk1 expression is seen exclusively in focal adhesions and has been shown to interact with nephrin [9]. Cells plated on laminin coated glass slips shows presence of phosphorylated nephrin in focal adhesion as well as co-localization of phospho-nephrin with activated β1 integrin (Fig 8B). Though the resolution offered by immunostaining in tissue sections is not high enough to conclusively prove the direct physical contact between integrin and nephrin. It is feasible that during development these two proteins are proximal enough to have a signaling relationship.

## Discussion

The need for ligation of Nephrin’s extracellular domain for its phosphorylation has been an important gap and controversy in the field of Nephrin biology. Both *in vivo* and *in vitro* studies suggest nephrin phosphorylation is diminished following binding to its extracellular ligand, nephrin itself or neph1 [6,9,11]. At the same time there are reports that nephrin is phosphorylated at its basal ligated state and decreases following injury [48,49]. There are few possible explanations for these divergent observations. Nephrin’s cytoplasmic domain has 10 tyrosine residues and there are likely to be differences in phosphorylation depending on the residue- specific phospho-antibody being used. Furthermore, we have observed differences in the way the sample is processed and the conditions used to unmask the epitopes. Incomplete extraction of phosphorylated nephrin can alter the observations as well. Phosphorylated nephrin is tightly bound to actin and is sequestered into the pellet following cell or tissue lysis. This requires extraction by reagents like potassium iodide for analysis [50,51]. It is well established that the Src family kinase Fyn is responsible for phosphorylation of nephrin on tyrosine residues that are necessary for the nephrin-nck interaction [6,12]. Here we present data that integrin ligation in cultured podocytes results in nephrin tyrosine phosphorylation. Although integrins are devoid of inherent catalytic activity, engagement of integrins by extracellular matrix ligands triggers ‘outside-in’ signaling that translates into specific cell behaviors [42,52]. A prominent consequence of integrin engagement or ligation is activation of Src and FAK family protein tyrosine kinases [19,53,54]. FAK activation was shown to occur by binding directly to the cytoplasmic domain of β1 integrin at focal adhesion sites [17,18]. Integrin β3 cytoplasmic tail was shown to interact with the Src Sh3 domain and competitively interfered with the natural, intramolecular interactions of c-Src SH3 domain and promotes increased c-Src activity [15]. Similarly, Fyn activation was observed in oligodendrocytes, where integrin-activation dependent down-regulation of Csk, a negative regulator of the Src family kinase was responsible for the increased Fyn activity [41].

The heterodimer formed between the various integrin subunits provides specificity to its binding with different ECM components (Fig 1A). The laminin receptor α3β1 is highly expressed integrin in kidneys and is found in both the glomeruli as well as the tubules. Global α3 integrin deleted mice have abnormalities in both the glomerulus as well as the collecting system [22]. Selective deletion of α3 in podocytes resulted in severe developemental abnormalities of the foot processes and masssive proteinuria [55]. Similar but a more severe phenotype was observed when the β1 subunit was deleted in podocytes suggesting that integrin α3β1 is the major integrin involved in podocyte homeostasis and glomerular development [27]. Furthermore, human mutations in the integrin α3 subunit is associated with severe renal abnormalities and premature death [56]. The two major collagen receptors α1β1 and α2β1 are also widely expressed in kidneys [57,58]. Neither of these integrins affect kidney development because integrin α1- and α2 null mice do not develop any obvious renal phenotype [59,60]. Intrestingly, pharmacological inhibition of α2β1 integrin activation resulted in amelioration of adriamycin induced glomerular injury [59].

Plating podocytes on surfaces coated with laminin or fibronectin resulted in nephrin tyrosine phosphorylation that was apparent within 15 minutes. In contrast, an increase in nephrin phosphorylation is not observed when cells were plated on collagen-coated or uncoated surface. There were differences in the duration and intensity of nephrin phosphorylation based on the ECM component. Though there was higher fold increase in nephrin phosphorylation at earlier time points when cells were plated on laminin there was persistence of nephrin phosphorylation for a longer time on fibronectin. The increase in nephrin phosphorylation, as well activation of integrin β1 was lower when podocytes were plated on collagen and uncoated surface. This heterogeneity in response to different matrix components suggests specificity of the proximal cue that is responsible for nephrin phosphorylation. At the same time there is some redundancy in the signaling pathway presumably to accommodate change in GBM composition during development. The abrogation of nephrin phosphorylation in the presence of β1 and β3 integrin blocking antibody suggests the importance of integrin activation for nephrin phosphorylation to occur. So far, fibronectin or RGD-binding integrin α5β1 has not been shown to play a role in kidney disaease. Expression of α5β1 integrin in our cultured podocytes might be a consequence of adapting to a non-physiological enviornment. Though we observed an increase in nephrin phosphorylation in response to fibronectin coated surface, this may not be as relevant *in vivo*. We were also able to show that Src kinase activity is necessary for integrin-dependent nephrin phosphorylation. There is increase in Src activity as evidenced by increase in Src Y416 phosphorylation following plating of cells on laminin coated surface. In the presence of blocking antibodies against both β1 and β3 integrin, both Src activation and nephrin phosphorylation is decreased. Additionaly, the abrogation of nephrin phosphorylation in SYF cells (Fig 7) supports the hypothesis that integrin-dependent Src activation might be responsible for the observed increase in nephrin phosphorylation.

**Fig 9:**
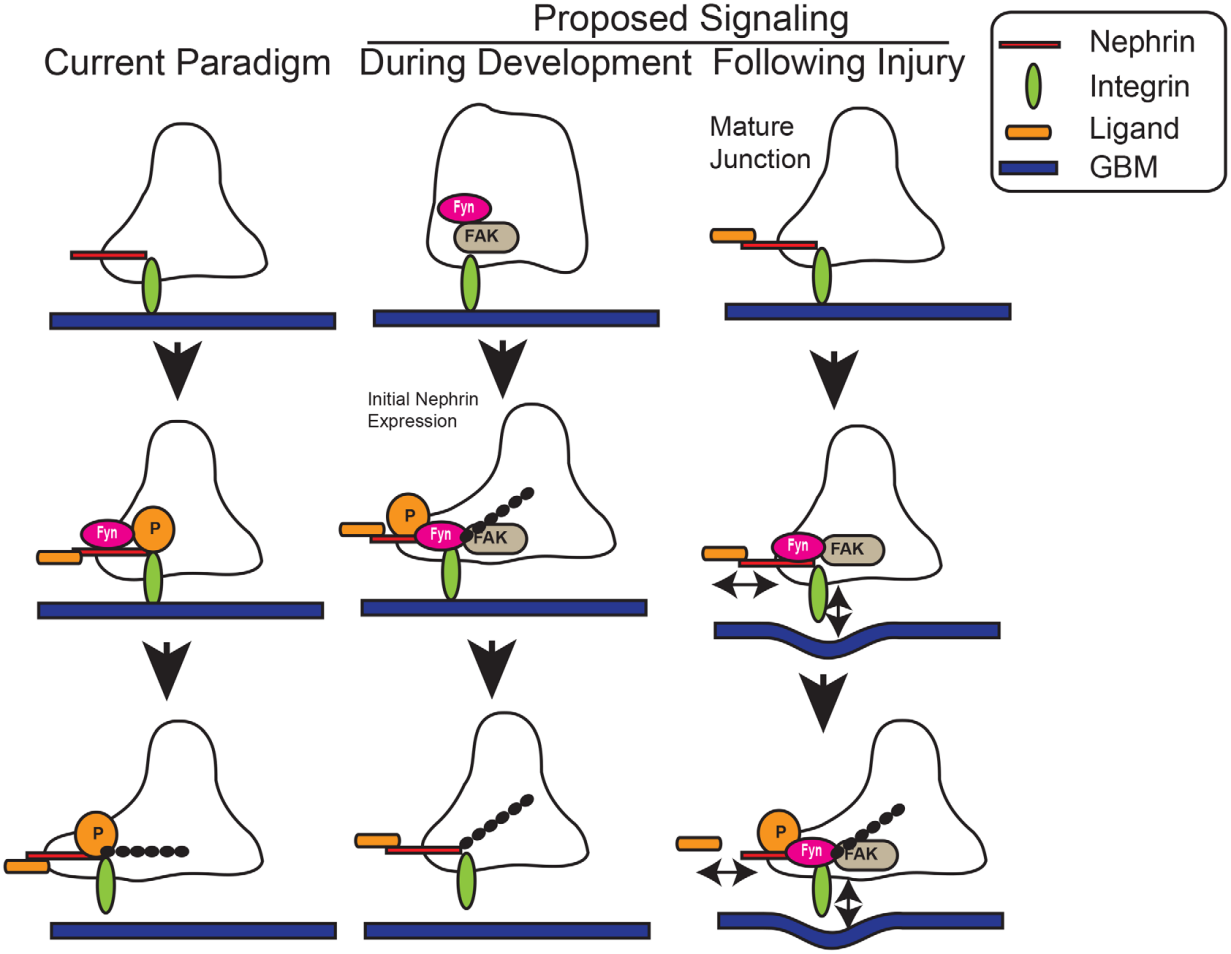
Schematic showing existing paradigm as well as proposed signaling that results in nephrin phosphorylation via integrin activation during development and following injury.

An argument can be made that our observations are made in cultured podocyes and may not represent events occuring *in vivo*. In cultured podocytes endogenous nephrin is seen at the leading edge as well as in focal adhesions. We observed co-localization of activated β1 integrin and phospho-nephrin by immunostaining both during development in newborn mouse kidney sections as well as in cultured podocytes. Based on the foot process structure it is possible that nephrin, focal adhesion complex and integrin are in close proximnity if not physically in contact with each other (see schematic in Fig 9). It has been observed that nascent podocyte foot processes arise simultaneous to initial nephrin expression [61]. It is plausbile that during development, nephrin’s arrival at the slit diaphragm in physical proximity to the integrin-focal adhesion complex results in nephrin phosphorylation followed by actin polymerization and membrane protrusion. Similar to podocyte-podocyte junction disruption, perturbation of the podocyte-GBM interface is likely to occur in podocyte injury. This would result in integrin activation, which may be responsible for an increase in nephrin phosphorylation observed following injury leading to cytoskeletal changes that are responsible for foot process spreading. In our recent publication, we showed that Shp2-mediated Src activation enhances nephrin phosphorylation [62]. Interestingly, integrin engagment has been shown to result in Shp2-mediated Src kinase Fyn activation [13,46]. Future studies to validate our observations in a more physiological setting are needed. Attempts to investigate the state of nephrin phosphorylation in mouse kidneys lacking β1 integrin was unsuccessful due to pre-existing podocyte injury and poor nephrin signal. It is difficult to demonstrate the signaling relation between integrin ligation and nephrin phosphorylation in existing models of podocyte injury as the signaling events that occur during injury are not well defined. To our knowledge there are no available reagents to selectively activate integrin signaling *in vivo*. Despite its limitation, our study is an important step forward in explaining the observations that have been made *in vivo* in regards to nephrin phosphorylation.
